# Protection against doxorubicin-induced myocardial dysfunction in mice by cardiac-specific expression of carboxyl terminus of hsp70-interacting protein

**DOI:** 10.1038/srep28399

**Published:** 2016-06-21

**Authors:** Lei Wang, Tian-Peng Zhang, Yuan Zhang, Hai-Lian Bi, Xu-Min Guan, Hong-Xia Wang, Xia Wang, Jie Du, Yun-Long Xia, Hui-Hua Li

**Affiliations:** 1 Department of Cardiology, Institute of Cardiovascular Diseases, First Affiliated Hospital of Dalian Medical University, Dalian 116011, China; 2 Department of Pathophysiology, School of Basic Medical Sciences, Baotou Medical College, Baotou 014060, China; 3 Department of Physiology and Pathophysiology, School of Basic Medical Sciences, Capital Medical University, Beijing 100069, China; 4 Beijing AnZhen Hospital the Key Laboratory of Remodeling-Related Cardiovascular Diseases, Capital Medical University, Beijing 100029, China; 5 Department of Nutrition and Food Hygiene, School of Public Health, Dalian Medical University, Dalian 116044, China

## Abstract

Carboxyl terminus of Hsp70-interacting protein (CHIP) is a critical ubiquitin ligase/cochaperone to reduce cardiac oxidative stress, inflammation, cardiomyocyte apoptosis and autophage etc. However, it is unclear whether overexpression of CHIP in the heart would exert protective effects against DOX-induced cardiomyopathy. Cardiac-specific CHIP transgenic (CHIP-TG) mice and the wild-type (WT) littermates were treated with DOX or saline. DOX-induced cardiac atrophy, dysfunction, inflammation, oxidative stress and cardiomyocyte apoptosis were significantly attenuated in CHIP-TG mice. CHIP-TG mice also showed higher survival rate than that of WT mice (40% versus 10%) after 10-day administration of DOX. In contrast, knockdown of CHIP by siRNA *in vitro* further enhanced DOX-induced cardiotoxic effects. Global gene microarray assay revealed that after DOX-treatment, differentially expressed genes between WT and CHIP-TG mice were mainly involved in apoptosis, atrophy, immune/inflammation and oxidative stress. Mechanistically, CHIP directly promotes ubiquitin-mediated degradation of p53 and SHP-1, which results in activation of ERK1/2 and STAT3 pathways thereby ameliorating DOX-induced cardiac toxicity.

Chronic heart failure is a consequence of cardiac remodeling processes that can be induced by various types of heart diseases such as myocardial infarction, chronic hypertension, or toxic agents. The anthracycline doxorubicin (DOX) is widely used as an effective anti-tumour drug, but its clinical use is limited by cardiotoxicity leading to congestive heart failure^1^,^2^. Although a variety of approaches to protect the heart against DOX-induced cardio-toxicity have been attempted, treatment to prevent short and long term DOX-induced cardiac damage remains limited^3^. Several lines of evidence support a critical role for molecular chaperones in the homeostasis of the cardiovascular system. Heat shock proteins are abundantly expressed within myocardial cells, and the inducible heat shock protein Hsp70 is upregulated after ischemic injury to the heart, and deletion of Hsp70 might induce cardiac dysfunction and development of cardiac hypertrophy^4^. Moreover, Hsp20 interacting with phosphorylated AKT reduces endotoxin- or DOX-induced oxidative stress and cardiotoxicity^5^,^6^. Importantly, *in vitro* screening for tetratricopeptide repeat (TPR)-containing proteins has identified carboxyl terminus of Hsp70-interacting protein (CHIP) as a novel TPR-containing proteins in the human heart. CHIP interacts with Hsp70/Hsp90 and regulates chaperone activity and protein quality control at multiple levels^7^.

CHIP is known to be a dual-function cochaperone/ubiquitin ligase that is highly expressed in the heart and other tissue cells. CHIP has ubiquitin ligase activity and targets chaperone-bound client proteins such as p53, tau, ErbB2, Ask1, Foxo1, and myocardin for the ubiquitin-mediated degradation^8^,^9^,^10^,^11^,^12^,^13^. Recently, CHIP was reported to play a critical role in cardioprotection during oxidative stress^14^. CHIP deficient mice result in markedly increased apoptosis in cardiomyocytes and endothelial cells after infarction injury^15^. In contrast, overexpression of CHIP inhibits ASK1- and p53-mediated apoptosis in cardiomyocytes and other cells^10^,^16^,^17^. However, the physiological *in vivo* role of CHIP overexpression in DOX-induced cardiac injury has not yet been investigated.

On the basis of previous findings, we therefore postulated that increased CHIP levels would ameliorate DOX-induced cardiotoxicity. To test this hypothesis, wild-type (WT) and CHIP transgenic mice (CHIP-TG) were administered with a single dose of DOX (20 mg/kg; i.p.) for 5 or 10 days. Cardiac function, histologic aspects, cytokine production, apoptosis, oxidative stress and survival were examined. Here we showed that cardiac-specific CHIP expression significantly improved cardiac function and prolonged survival *in vivo* by blocking DOX-induced apoptosis, inflammation and oxidative stress. The cardioprotective effects of CHIP against DOX toxicity were associated with ubiquitin-mediated degradation of p53, SHP-1 and preserved activation of ERK1/2 and STAT3 signaling pathways. These results suggest that CHIP may be a potential therapeutic target for the treatment of DOX-induced heart failure.

## Results

### DOX downregulates the expression of CHIP in neonatal rat cardiomyocytes and in the mouse heart

To determine the functional role of cardiac CHIP in response to DOX treatment, we first examined the expression of CHIP, HSP70 and HSP90 in neonatal rat cardiomyocytes with different doses of DOX as indicated. Western blot analysis showed that DOX treatment markedly decreased CHIP expression in a dose-dependent manner, whereas no significant change in HSP70 and Hsp90 expression was observed (see Supplementary Fig. S1a). Moreover, injection of DOX (20 mg/kg; i.p.) in mice significantly decreased CHIP expression compared with control mice (see Supplementary Fig. S1b,c). These results indicate that CHIP expression is reduced in cardiomyocytes in response to DOX.

### Overexpression of CHIP improves DOX-induced cardiac dysfunction and mortality of mice

**Figure 1:**
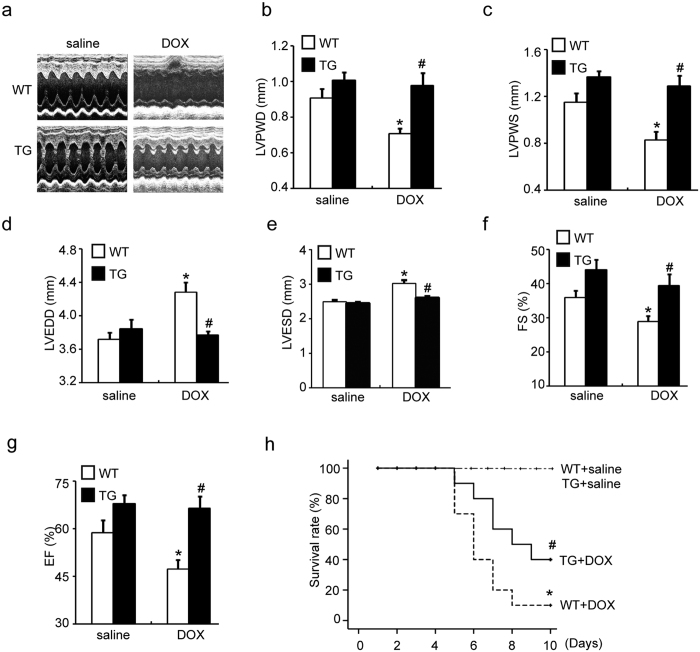
Cardiac specific overexpression of CHIP improves DOX-induced cardiac dysfunction and mortality of mice. (**a**) Representative M-mode echocardiograms of WT and CHIP-TG mice treated with saline or DOX. Quantitative group data for echocardiographic measurements: (**b**) LVPWD; (**c**) LVPWS; (**d**) LVEDD; (**e**) LVESD; (**f**) FS% and (**g**) EF% (n = 10 per group). (**h**) Ten-day survival for WT and CHIP-TG mice treated with a single dose of DOX (20 mg/kg). Survival was analyzed by the Kaplan-Meier approach and the log-rank test (n = 20 per group). *P < 0.05 versus WT + saline group, ^#^P < 0.05 versus WT + DOX group. LVPWD: left ventricular posterior wall thickness at end-diastole; LVPWS: left ventricular posterior wall thickness at end-systole; LVEDD: left ventricular end diastolic dimensions; LVESD: left ventricular end systolic dimensions; FS: fractional shortening; EF: ejection fraction.

To evaluate whether increased CHIP expression protects against DOX-induced cardiac dysfunction, CHIP-TG mice were injected with a single dose of DOX (20 mg/kg) for one time. Five days after DOX injection, cardiac function was evaluated by echocardiography. Figure 1a showed representative echocardiograms after vehicle or DOX administration in WT and CHIP-TG mice (Fig. 1a). WT mice were found to exhibit a significant decrease of left ventricular posterior wall thickness at end-diastole (LVPWD) and left ventricular posterior wall thickness at end-systole (LVPWS), a markedly increase of the left ventricular end diastolic dimensions (LVEDD) and left ventricular end systolic dimensions (LVESD), whereas these alterations were markedly restored in CHIP-TG mice (Fig. 1b–e). Moreover, DOX treatment caused a pronounced reduction in cardiac contractility reflected by fractional shortening (FS%) and ejection fraction (EF%) in WT mice, and this effect was significantly attenuated in CHIP-TG mice (Fig. 1f,g). In addition, the survival rate was significantly higher in CHIP-TG than in WT mice after DOX treatment (40% versus 10%) (Fig. 1h). No death was observed in saline-treated groups (Fig. 1h). Together, these results indicate that overexpression of CHIP ameliorates cardiac functional deterioration and survival of mice in response to DOX.

### Cardiac CHIP overexpression protects DOX-induced cardiac injury, apoptosis, atrophy, inflammation and oxidative stress in mice

**Figure 2:**
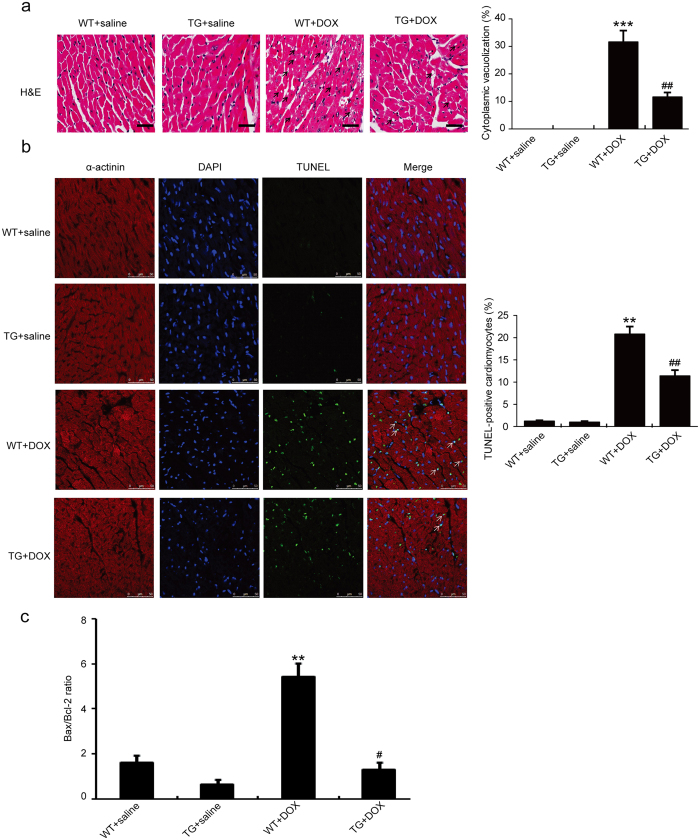
CHIP overexpression attenuates DOX-induced cardiac injury and cardiomyocyte apoptosis. (**a**) Representative H&E staining of heart sections on day 5 after saline or DOX treatment (n = 6 per group). Arrows indicate representative vacuolization. Quantitative results of cardiomyocyte vacuolization were shown on the right panel (scale bar = 50μm). (**b**) Representative photomicrographs of cardiomyocyte apoptosis in myocardium examined by TUNEL assay (scale bar = 50 μm). Quantitative results of TUNEL-positive cardiomyocytes from WT and CHIP-TG mice were shown on the right (n = 6 per group). (**c**) qPCR analysis of Bax and Bcl-2 mRNA expression in WT and CHIP-TG mice treated with saline or DOX (n = 6 per group). **P < 0.01, ***P < 0.001 versus WT + saline mice; ^#^P < 0.05, ^##^P < 0.01 versus WT + DOX mice.

**Figure 3:**
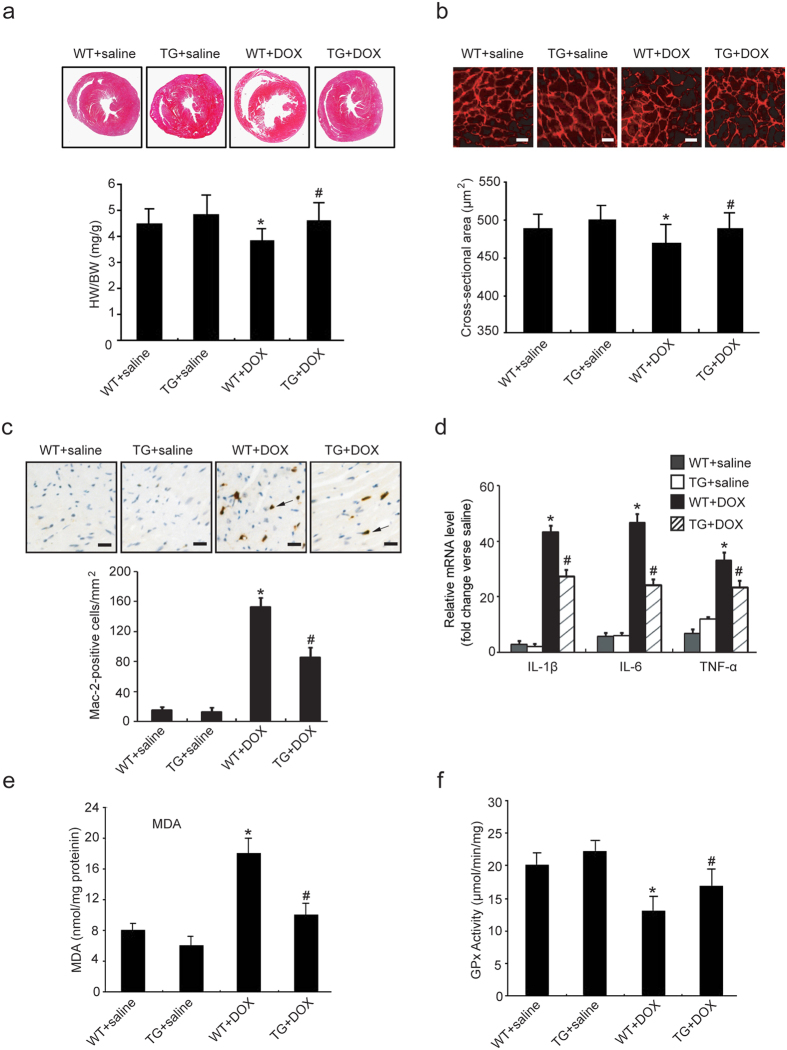
CHIP overexpression inhibits DOX-induced cardiac atrophy, inflammation and oxidative stress. (**a**) The representative H&E staining of heart sections on day 5 after saline or DOX treatment of each group were shown above. The ratio of heart weight to body weight (HW/BW) in WT and CHIP-TG mice were shown below (n = 6 per group). (**b**) Heart sections were stained with wheat germ agglutinin-TRITC conjugate (red) to determine the myocyte fiber diameter (top; scale bar = 50 μm). Quantitative results of cardiomyocyte cross-sectional areas from the indicated groups (bottom, n = 6 per group). (**c**) Representative immunohistochemical staining with anti-Mac-2 antibody in the heart sections. (top, scale bar = 50 μm). Quantitative analysis of Mac-2-positive cells in each group (bottom, n = 6 per group). (**d**) qPCR analysis of mRNA expression of IL-1β, IL-6 and TNF-α was performed (n = 6 per group). (**e**,**f**) Malondialdehyde (MDA) levels and glutathione peroxidase (GPX) activities were measured to estimate the extent of lipid peroxidation and anti-oxidation in heart homogenates from WT and CHIP-TG mice treated with saline or DOX (n = 6 per group). *P < 0.05 versus WT + saline mice; ^#^P < 0.05 versus DOX-treated WT mice.

To test whether CHIP overexpression inhibits DOX-induced cardiac injury and apoptosis *in vivo*, we first examined heart section with H&E staining and TUNEL assay. DOX-treated WT mice exhibited marked focal cytoplasmic vacuolization (~31.7%), a hallmark of cardiomyocyte injury, which is consistent with previous reports^18^, whereas this effect was significantly reduced in CHIP-TG mice (~11.7%) (Fig. 2a). TUNEL-positive cardiomyocytes were barely detectable in the heart of mice with saline injection. DOX injection significantly increased the number of TUNEL-positive cardiomyocytes in WT mice (~20.9%). Conversely, this change was markedly attenuated in CHIP-TG mice (~11.4%) (Fig. 2b). In addition, DOX treatment resulted in a significant increase in Bax/Bcl-2 ratio in WT mice but not in CHIP-TG mice (Fig. 2c). Furthermore, we found that DOX injection significantly reduced the wall thickness and increase the diameter of ventricular. The ratio of heart weight/body weight (HW/BW) and cross-sectional area of cardiomyocytes were also decreased compared with saline group, indicating a ventricular dilation after DOX treatment. In contrast, these changes were reversed in CHIP-TG mice (Fig. 3a,b). Five days after DOX injection, the number of Mac-2-positive macrophages and the expression levels of IL-1β, IL-6 and TNF-α mRNA were significantly decreased in CHIP-TG hearts compared with WT hearts (Fig. 3c,d). In addition, malondialdehyde (MDA) level was significantly decreased but glutathione peroxidase (GPx) activity was markedly increased in CHIP-TG mice compared with WT mice (Fig. 3e,f). There was no difference in the number of infiltrated macrophages, the levels of the cytokines, the MDA level and GPx activity between two group hearts after saline injection (Fig. 3c–f).

### Knockdown of CHIP by siRNA increases DOX-induced apoptosis, inflammatory response and oxidative stress in neonatal rat cardiomyocytes

**Figure 4:**
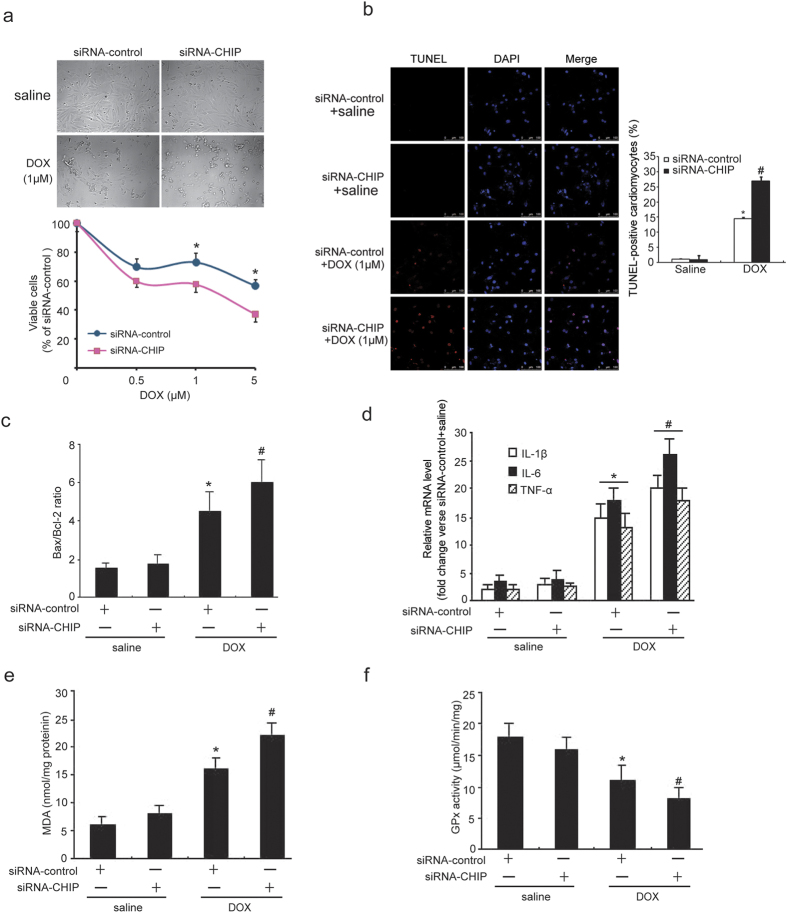
Knockdown of CHIP enhances DOX-induced cell apoptosis, pro-inflammatory cytokine expression and oxidative stress in neonatal rat cardiomyocytes. (**a**) Cardiomyocytes were infected with adenovirus siRNA-control or siRNA-CHIP and then treated with different doses of 0.5, 1 or 5 μM DOX for 24 hours. Cell viability was measured by trypan blue exclusion assay (top, scale bar = 100 μm). Quantitative analysis of viable cells (bottom). (**b**) Apoptosis was detected by TUNEL assay 24 hours after DOX (0.5 μM) treatment (left, scale bar = 50 μm). Quantitative analysis of TUNEL-positive cells (right). (**c**) qPCR analysis of Bax and Bcl-2 mRNA expression was performed, and Bax/Bcl-2 ratio was determined for each group. (**d**) qPCR analysis of proinflammtory cytokines IL-1β, IL-6, TNF-α mRNA expression was performed. (**e**,**f**) MDA levels and GPx activities were measured in cardiomyocytes homogenates from siRNA-control and siRNA-CHIP infected groups. *P < 0.05 versus siRNA-control + saline. ^#^P < 0.05 versus siRNA-control + DOX.

To further confirm the role of CHIP knockdown in DOX-triggered cardiac injury, the expression of endogenous CHIP was reduced in neonatal rat cardiomyocytes by infection of adenovirus siRNA-CHIP or siRNA-control. The infection efficiency reached more than 95% after 24 hours. Cardiomyocyte viability was significantly lower and the number of TUNEL-positive cell was higher in siRNA-CHIP-infected group than siRNA-control (Fig. 4a,b). qPCR analysis revealed that DOX treatment resulted in an increase in the Bax/Bcl-2 ratio, the expression of pro-inflammatory cytokines (IL-1β, IL-6 and TNF-α) and the MDA level and a decreased in the GPx activity in siRNA-control than that in untreated group, and these effects were further enhanced in siRNA-CHIP-infected cells in response to DOX (Fig. 4c–f). No difference in these alteration was observed under the basal condition (Fig. 4).

### CHIP overexpression attenuates DOX-induced cardiac injury by regulating multiple mechanisms in the heart

**Figure 5:**
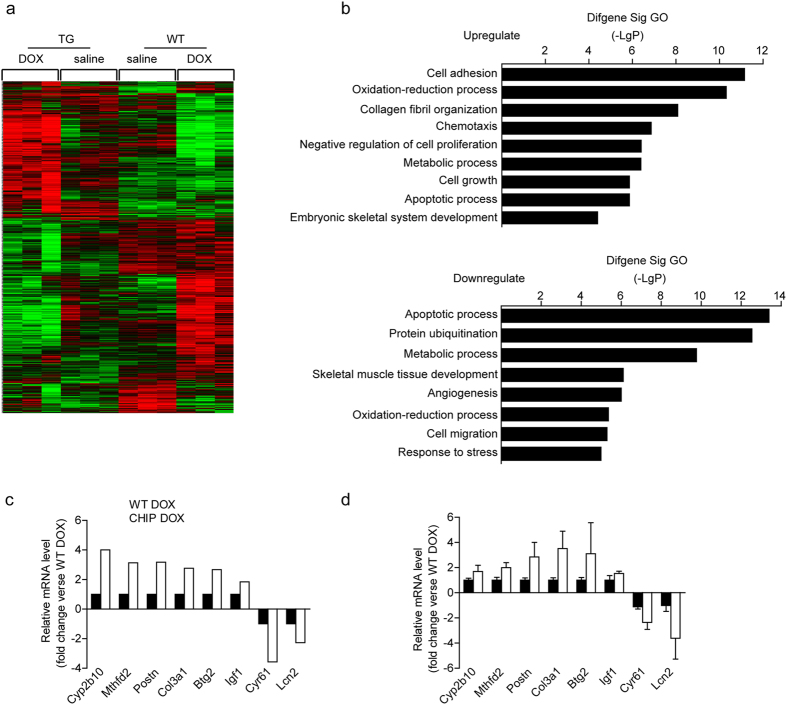
Microarray assay in WT and CHIP-TG hearts after DOX treatment. (**a**) Hierarchical clustering depicting expression profiles of differentially expressed genes in WT and CHIP-TG mice on day 5 after saline/DOX treatment (n = 3 per group). Gene expression levels are shown as color variations (red, high expression; green, low expression). (**b**) The significant different GO of up-regulated (above) and down-regulated (below) between WT and CHIP-TG mice-treated with DOX. (**c**) The primary microarray data showed fold change of the selected genes expression in WT and CHIP-TG mice-treated with DOX. (n = 6 per group). (**d**) qPCR validation for the selected genes mRNA levels in WT and CHIP-TG mice-treated with DOX. (n = 6 per group).

To investigate the molecular events of cardiac contraction improvement in CHIP-TG mice after DOX injection, we performed microarray assay to examine the effects of CHIP overexpression on the global gene expression profile of hearts after saline and DOX injection. We found that cardiac CHIP overexpression resulted in significant regulation of 1938 genes compared with WT mice after DOX-treatment. Among them, 657 genes were significantly upregulated, and 1281 genes markedly downregulated. The hierarchical clustering analysis showed differentially expressed genes in the four groups (Fig. 5a). We then analyzed the functional bias of these differentially expressed genes according to Gene Ontology (GO) classifications. A total of 511 GO items associated with up-regulated genes and 477 GO items associated with down-regulated genes were significantly altered between CHIP-TG and WT mice after DOX injection. Moreover, the majority of enriched GO terms among the differentially expressed genes were associated with apoptosis, immune/inflammation, cell growth and oxidative stress (Fig. 5b, Table 1). Some of the significantly up-regulated genes such as Cyp2b10, Mthfd2, Postn, Col3a1, Btg2, Igf1, and down-regulated genes such as Cyr61 and Lcn2 were also verified by qPCR analysis (Fig. 5c,d). Furthermore, analysis of pathways of genes with significantly differences between CHIP-TG and WT mice showed that 113 pathways were significantly up-regulated and 140 pathways were down-regulated, which are mainly involved in regulating metabolic pathways, cytokine-receptor interaction, p53, insulin signaling, mTOR, MAPKs, and ubiquitin-mediated proteolysis, etc. (see Supplementary Fig. S3).

### Effect of CHIP on the p53, ERK and STAT3 signaling pathways

**Figure 6:**
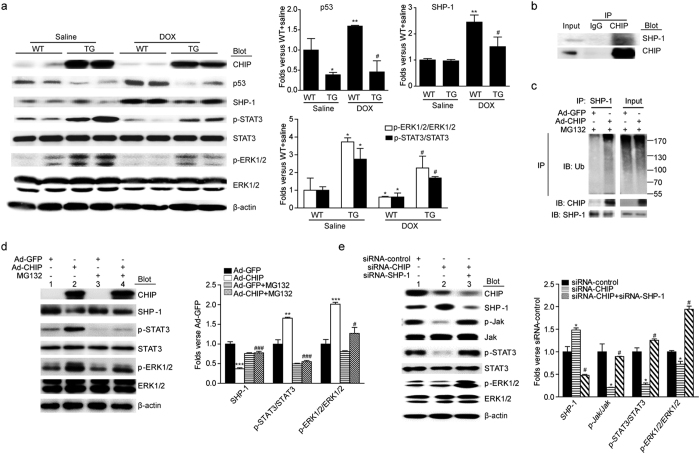
CHIP degrades SHP-1 through ubiquitin-proteasome system and activates STAT3 and ERK1/2 signaling pathway. (**a**) Western blot analysis of protein levels of p53, SHP-1, p-STAT3/STAT3 and p-ERK1/2/ERK1/2 in WT and CHIP-TG hearts (left). Quantitative analysis of relative protein levels (right). *P < 0.05, **P < 0.01 versus WT mice + saline; ^#^P < 0.05 versus WT mice + DOX. (**b**) The interactions of SHP-1 with CHIP were detected with co-immunoprecipitation in H9c2 cells. H9c2 cells lysates were immune-precipitated with anti-CHIP antibody or control IgG, and the immune-precipitates were subjected to sodium dodecyl sulfate-polyacrylamide gel electrophoresis (SDS-PAGE) and immunoblotted with anti-SHP-1 antibody. (**c**) Equal amounts of lysates from Ad-GFP/Ad-CHIP adenovirus H9c2 cells were immune-precipitated with SHP-1 antibody and analyzed by immunoblotted with anti-ubiquitin antibody to detect ubiquitylated forms of SHP-1 *in vitro*. (**d**) Western blot analysis of protein levels of SHP-1, p-STAT3/STAT3 and p-ERK1/2/ERK1/2 in H9c2 cells after transfected with GFP/CHIP adenovirus and treated with or without MG 132 (left). Quantitative analysis of relative protein levels (right). (**e**) Western blot analysis of protein levels of SHP-1, p-Jak/Jak, p-STAT3/STAT3 and p-ERK1/2/ERK1/2 in H9c2 cells after transfected with siRNA-control, siRNA-CHIP or siRNA-SHP-1 (left). Quantitative analysis of relative protein levels (right). *P < 0.05, **P < 0.01, ***P < 0.001 versus Ad-GFP/siRNA-control; ^#^P < 0.05, ^###^P < 0.001 versus Ad-GFP + MG132/siRNA-CHIP.

To further determine which signaling pathways were involved in the possible mechanism of CHIP cardioprotection against DOX, we selectively assessed the activation of several major signaling pathways, including p53, IGF1R/AKT, MAPKs (ERK, JNK) and gp130/STAT3, which play important roles in regulating cardiac hypertrophy, myocyte apoptosis, inflammation, and oxidative stress^19^. We found that DOX-treatment did not significantly reduce the protein levels of receptors IGF1R and gp130, but markedly increased the levels of p53 and phosphorylated JNK1/2, decreased the levels of phosphorylated AKT, ERK1/2, Jak and STAT3 in WT hearts compared with saline-treated group, whereas the levels of p53 and phosphorylated ERK1/2, Jak and STAT3 rather than phosphorylated AKT and JNK1/2 were significantly reversed in CHIP-TG mice (Fig. 6a and Supplemental Fig. S2a,b). In contrast, knockdown of CHIP by siRNA markedly increased the level of p53 protein and decreased ERK1/2 and STAT3 phosphorylation in neonatal rat cardio-myocytes compared with DOX-treated siRNA-control (see Supplementary Fig. S2c). These results suggest that the protective effect of CHIP on DOX-induced cardiotoxicity was partially mediated by p53, ERK1/2 and STAT3 signaling pathways.

### CHIP promotes ubiquitin-mediated degradation of SHP-1

Previous study have proved that CHIP was responsible for p53 degradation in the heart^20^. To investigated how CHIP mediates activation of Jak/STAT3 and ERK1/2, we first assessed whether CHIP affected the tyrosine phosphatase SHP-1 that was known to suppress Jak/STAT3 and ERK1/2 pathways in H9c2 cells^21^,^22^. Our co-immunoprecipitation assays revealed that SHP-1 were precipitated by antibody against CHIP, but not by control rabbit IgG (Fig. 6b), indicating that CHIP directly interacted with SHP-1 in cardiomyocytes. We then sought to determine whether CHIP downregulates SHP-1 in heart tissues, and found that the SHP-1 protein level was significantly increased in DOX-treated wild-type mice, whereas this effect was abrogated in CHIP-Tg hearts (Fig. 6a). To establish whether CHIP mediated SHP-1 ubiquitylation, we immuneprecipitated SHP-1 from Ad-GFP- or Ad-CHIP-infected H9c2 cells and immunoblotted for ubiquitylated species. We found that increased expression of CHIP significantly increased SHP-1 ubiquitylation compared with Ad-GFP control (Fig. 6c). To further determine whether CHIP promotes SHP-1 degradation through proteasomes, we used proteasome inhibitor MG132 to treat H9c2 cells. Consistent with the results from animal experiments, overexpression of CHIP also significantly decreased the protein levels of SHP-1 compared with Ad-GFP control (Fig. 6d, lane 2 vs 1), and this effect was markedly reversed MG132 (Fig. 6d, lane 4 vs 2), indicating that CHIP targets SHP-1 protein for proteasome degradation. To further study the crucial role of CHIP in regulating SHP-1 degradation and activation of Jak/STAT3 and ERK1/2 pathways, H9c2 cells were transfected with siRNA-CHIP, siRNA-SHP-1 or siRNA control. We found that knockdown of CHIP significantly upregulated SPH1 protein level but decreased the phosphorylation of Jak, STAT3 and ERK1/2 (Fig. 6e, lane 2 vs 1), whereas depletion of SHP-1 markedly reversed this effect (Fig. 6e, lane 3 vs 2), indicating that CHIP promotes Jak/STAT3 and ERK1/2 activation through degradation of SHP-1.

## Discussion

CHIP, as a chaperone and ubiquitin E3 ligase, has been widely explored in protection against ischemic injury and other stress stimuli^7^,^10^,^13^,^14^,^15^,^17^,^18^. However, its possible protective effects on DOX-induced cardio-toxicity and underlying mechanisms are not well defined. In this report, we provide the first evidence that CHIP *in vivo* and *in vitro* protects against DOX-induced cardiac apoptosis, atrophy, inflammatory and oxidative stress resulting in prevention of cardiac dysfunction and improvement of mouse survival. These effects were associated with alteration of multiple signaling pathways, including decreased p53 and increased activation of ERK1/2 and STAT3 signaling pathways.

DOX is one of the most important anticancer agents. However, clinical use of DOX is limited by its cardiotoxicity^1^,^2^,^3^. Although the precise mechanisms whereby DOX induces myocardial injury have not been fully elucidated, it is widely accepted that the DOX induces cardiac injury via several mechanisms, including activation of ubiquitin-proteasome system, sarcomere reorganization, induction of proinflammatory cytokines, free radical generation and apoptotic cell death that are the typical changes in DOX-induced heart failure^23^. Several recent findings indicate that cardiomyocyte apoptosis is a leading cause of cardiac dysfunction in DOX-induced cardiomyopathy^23^. DOX evokes oxidative stress and expression of pro-apoptotic protein p53 and Bax, which activates apoptotic signaling leading to cardiomyocyte apoptosis in the heart and in isolated cardiomyocytes^3^,^24^,^25^. Moreover, overexpression of antioxidant genes such as manganese superoxide dismutase and catalase in the heart protects mice against DOX-induced heart failure^26^,^27^. CHIP can activate HSF1 and protect against apoptosis and cellular stress^14^. CHIP also promotes ASK1 degradation leading to inhibition of cell apoptosis^10^. In contrast, CHIP knockout aggregates ischemia/reperfusion-induced myocardial apoptosis and dysfunction^15^,^16^. To seek evidence of the protective effect of CHIP in DOX-induced apoptosis, we conducted a series of TUNEL assays, analyses of Bax and Bcl-2 expression and measurement of oxidative stress *in vivo* and *in vitro*. Our results demonstrated that increased CHIP expression markedly preserved cardiac dysfunction and mouse survival (Fig. 1), effectively decreased TUNEL-positive cardiomyocytes, Bax/Bcl-2 ratio, MDA level but enhanced the activity of antioxidant enzyme GPx (Figs 2 and 3). These results suggest that CHIP may play a critical role in protecting cardiomyocyte apoptosis evoked by DOX partially through inhibition of oxidative stress.

Studies have demonstrated that DOX induces cardiac dysfunction, which was accompanied by marked cardiac atrophy^28^,^29^ and infiltration of inflammatory cells^30^. It has been reported that DOX can lead to cardiac atrophy^30^,^31^, which was also confirmed in our study (Fig. 3a,b). Our new finding is that cardiac-specific overexpression of CHIP exerts an anti-atrophic effect on heart caused by DOX (Fig. 3a,b). Oxidative stress can directly trigger cytokine expression, which was markedly increased in the early time and 5 days after DOX injection^32^. Recently, our results indicate that CHIP is involved in Ang II-induced expression of pro-inflammatory cytokines through inactivation of NF-κB in the mouse heart and neonatal rat cardiomyocytes^18^. Consistent with these observations, the present results showed that DOX-induced accumulation of macrophages and the expression of pro-inflammatory cytokines such as IL-1β, IL-6 and TNF-α were markedly suppressed in CHIP-TG mice (Fig. 3c,d). In contrast, knockdown of CHIP had opposite effect (Fig. 4).

Cardiac injury including apoptosis, atrophy, inflammation and oxidative stress induced by DOX ultimately leads to cardiomyopathy and congestive heart failure^1^. The mechanisms leading to DOX-induced myocardial damage may involve multiple signaling pathways, including p53, IGF1R/AKT, NF-kB, MAPKs (ERK, JNK), and gp130/Jak/STAT3^23^. For example, the p53 null mice show reduced cardiomyocyte apoptosis and concomitant improvements in cardiac function^33^. Inhibition of AKT and ERK pathways is associated with DOX-induced cardiotoxicity, which is prevented by the administration of cardio-protective reagents, such as oleylethanolamide, dexrazoxane, granulocyte colony-stimulating factor (G-CSF) that increased ERK1/2 activity^5^,^19^,^34^. Moreover, several studies have demonstrated that CHIP interacts with HSP90 and can regulate both ERK1/2 and Jak/STATs pathways in various cell types^35^,^36^,^37^,^38^,^39^,^40^,^41^. To further explore the protective mechanisms of CHIP in DOX-induced cardiac injury, we first performed microarray assay, and identified several signaling pathways were associated with cardiac injury, including ECM-receptor interaction, cytokine-receptor interaction, p53, insulin signaling, MAPKs, and ubiquitin-mediated proteolysis (Supplementary Fig. S3). Western blot further demonstrated that CHIP did not affect IGF1R/AKT, JNK1/2, gp130 (Supplementary Fig. S2A,B), but can interacted with and promoted SHP-1 degradation by proteasome thereby leading to activation of Jak/STAT3 and ERK1/2 (Fig. 6). A study has demonstrated that CHIP plays a role in mediating p53 degradation in the ischemic heart^20^. Gp130/Jak/STAT3 signaling pathway also participates in cardiac injury. Overexpression of STAT3 in the heart protects against DOX-induced cardiomyopathy, thus resulting in an improved survival rate by preventing progression of heart failure^42^. SHP-1 is known to serve as an important phosphatase of the Jak/STAT signaling pathway^21^,^43^,^44^. Loss of SHP-1 enhances the stability of Jak3 and activates Jak3/STAT3 signaling^21^. In addition, SHP-1 also interacts with ERK1/2 and negatively regulated MAPK/ERK1/2 signaling pathway 22,45,46. Together, above data indicate that CHIP protects against DOX-induced cardiac injury at least in part through ubiquitin-mediated degradation of p53 and SHP-1, and activation of Jak/STAT3 and ERK1/2 pathways.

In conclusion, this study demonstrates that cardiac-specific overexpression of CHIP ameliorated DOX-induced cardiac injury and dysfunction. The mechanisms underlying its protection at least in part were associated with the attenuation of DOX-triggered oxidative stress, the stability of p53 and SHP-1 proteins, and the activation of ERK1/2 and Jak/STAT3 signaling pathways, leading to inhibition of DOX-induced cardiomyocyte apoptosis, atrophy and inflammation. Consequently, left ventricular contractile function and mouse survival was improved after DOX injury. Thus, increased CHIP expression might be a promising therapeutic target for the treatment of DOX-triggered cardiac injury and heart failure.

## Materials and Methods

### Antibodies and reagents

Dulbecco’s modified Eagle’s medium, medium supplements, and fetal bovine serum was purchased from Invitrogen (Carlsbad, CA). Doxorubicin (DOX) was obtained from Sigma-Aldrich. Antibodies were purchased from Cell Signaling Technology (Beverly, MA) and (Abcam, Inc. (Cambridge, UK) (Supplemental Materials and methods).

### Cell culture and adenoviral constructs

Neonatal rat cardiomyocytes were isolated by enzymatic disassociation of 1-day-old Sprague Dawley rats with a mixture of 80 mg/kg ketamine and 30 mg/kg xylazine as described^47^. H9c2 cells were obtained from American Type Culture Collection (ATCC, Rockville, MD, USA). Recombinant adenoviruses expressing GFP alone (Ad-GFP), CHIP (Ad-CHIP), scrambled siRNA (siRNA-control, 5′-GTGCGTTGCTAGTACCAAC-3′; siRNA-CHIP, 5′-TGAGGCCAAGCACGATAAA-3′; siRNA-SHP-1, 5′-CCGCUACAAGAACAUUCUUTT-3′) driven by the cytomegalovirus promoter were generated with the Ad-Easy system^47^. Twenty-four hours after plating, cells were infected with Ad-GFP, Ad-CHIP, siRNA-control and siRNA-CHIP for 24 hours and then treated with 0.5 μM DOX for the indicated time points.

### Animals and treatments

Cardiac-specific CHIP transgenic mice were created as described previously^18^. CHIP transgenic (TG) mice (male, C57BL/6, 10–12-week-old) and the wild-type (WT) littermates and were randomly assigned to either the control group or the DOX-treated group. DOX was dissolved in saline and administered by intra-peritoneal injection (i.p) at a single dose of 20 mg/kg for one time^48^. Five days after DOX injection, we sacrificed mice and performed additional experiments. Control mice received injections of saline of comparable volume. Hearts were removed from mice anesthetized with a mixture of 80 mg/kg ketamine and 30 mg/kg xylazine intra-peritoneally. All procedures were approved by the Institutional Animal Care and Use Committee of Dalian Medical University. The investigation conforms to the Guide for the Care and Use of Laboratory Animals published by the US National Institutes of Health (NIH Publication No. 85-23, revised 2011).

### Echocardiography

Echocardiographic measurement was performed with a Vevo 770 ultrasound system (Visual Sonics Inc.) equipped with a 30-MHz transducer as described^48^. (Supplemental Materials and methods).

### Histological and immunohistochemical analysis

Histological analyses of hearts from WT and CHIP-TG mice were performed according to standard protocols. Heart sections were stained with hematoxylin and eosin (H&E), Masson’s trichrome and wheat germ agglutinin–TRITC conjugate as described previously^48^. Macrophage populations in the heart sections were detected by with anti-Mac-2 antibody or isotype control.

### Cell viability and TUNEL assay

Cell viability was determined by Trypan blue exclusion assay as described^48^,^49^,^50^. Cardiomyocte apoptosis was evaluated by the Dead End^TM^ Fluorometric TUNEL System (Promega) according to manufacturer’s instructions^51^. In the heart sections, cardiomyocytes were identified with α-actinin immunostaining, and nuclei were counterstained with DAPI. The percentage of TUNEL-positive cardiomyocytes was determined by counting 10 random fields per section under a microscope (magnification, x400).

### RNA analysis

Hearts from WT and CHIP-TG mice were excised, rinsed in PBS, frozen in liquid nitrogen. Total RNA was extracted by the Trizol reagent method (Invitrogen). The levels of Bax, Bcl-2, interleukin 1 β (IL-1β), IL-6, tumor necrosis factor α (TNF-α), and other selected gene mRNA expression were measured by quantitative real-time PCR (qPCR) with an iCyclerIQ system (Bio-Rad, USA)^18^. (Supplemental Materials and methods, Table 1).

### Measurement of maleic dialdehyde and glutathione peroxidase

Maleic dialdehyde (MDA) as indicator of lipid peroxidation^52^ was measured using the commercially available colorimetric assay kit (Nanjing Jiancheng Bioengineering Institute, China). Antioxidant enzyme was measured by Glutathione Peroxidase (GPx) Assay Kit (Calbiochem). Detailed operation procedure according to manufacturer’s instructions as described previously^48^.

### Microarray gene expression analysis

Total RNA was extracted with Trizol reagent (Invitrogen) from the hearts of saline and DOX-treated WT and CHIP TG mice (n = 3 per group) at day 5 after injection. The microarray assay was performed as described previously^51^,^53^,^54^. The bioinformatics of Gene Ontology (GO) and Pathway analysis were performed by using the Capital Bio Molecule Annotation System plate analysis as described before^55^. The gene expression data are available at the Gene Expression Omnibus (GEO) website: http://www.ncbi.nlm.nih.gov/geo/ (accession number GSE59672) (Supplemental Materials and methods).

### Western blot analysis

Protein samples were extracted from neonatal rat cardiomyocytes, or heart tissues which are collected on the fifth day after DOX injection. Western blot analyses were performed using indicated primary antibodies as described previously^18^. (Supplemental Materials and methods).

### Immunoprecipitation

Immunoprecipitations were performed as described before^47^. Briefly, H9c2 cells were maintained in 10% DMEM complete medium. Cell lysates were immunoprecipitated with anti-CHIP or anti-IgG antibody for 2 h at 4 °C, then beads were washed and analyzed by immunoblotting with anti-SHP-1 and anti-CHIP antibodies.

### Ubiquitination Assays

H9c2 cells were transfected with Ad-GFP and Ad-CHIP adenovirus for 24 h in serum-free medium. Then treated with 0.5 μM DOX/saline for 24 h in 10% DMEM complete medium. Cell lysates were immunoprecipitated with anti-SHP-1 antibody, and analyzed by immunoblotting using anti-ubiquitin, anti-SHP-1 and anti-CHIP antibodies as previously described^47^.

### Statistical analysis

Data are presented as means ± SEM. Differences between WT and CHIP-TG mice were evaluated for statistical significance using Student’s t test or by two-way ANOVA. Survival curves after DOX injection (20 mg/kg; i.p.) were created in WT (n = 20) and CHIP-TG (n = 20) mice by the Kaplan–Meier method and the log-rank test. The two-side Fisher’s exact test and χ^2^ test were used to classify the GO and Pathway category, and FDR was calculated to correct the P-value. A P < 0.05 was regarded as significant.

## Additional Information

**How to cite this article**: Wang, L. *et al.* Protection against doxorubicin-induced myocardial dysfunction in mice by cardiac-specific expression of carboxyl terminus of hsp70-interacting protein. *Sci. Rep.* **6**, 28399; doi: 10.1038/srep28399 (2016).

## Supplementary Material

Supplementary Information

## Figures and Tables

**Table 1:** Genes Dys-regulated in TG Mice vs WT Controls Five Days After DOX-treatment.

Upregulated (Fold Increase)	Downregulated (Fold Increase)
**Apoptosis**	
Btg2 (2.67)	Lcn2 (2.27)
Phlda3 (2.52)	Hmox1 (2.04)
Dnase2a (2.11)	Plscr1 (1.72)
Asns (2.09)	Mical1 (1.72)
Mmp2 (2.08)	Nr4a1 (1.67)
Cxcl12 (2.05)	Zfp110 (1.61)
Sh3kbp1 (1.99)	Malt1 (1.56)
Igf1 (1.85)	Bcl2l1 (1.54)
Slit2 (1.78)	Mknk2 (1.54)
Chac1 (1.75)	Ntn1 (1.54)
Pla2g4a (1.73)	Casp9 (1.52)
Aen (1.72)	Dapk1 (1.52)
Bcl2 (1.62)	Xrcc5 (1.52)
Sfrp1 (1.62)	Bik (1.52)
Ccng1 (1.60)	
Grk5 (1.59)	
Ptgfr (1.58)	
Fhl2 (1.57)	
Atf5 (1.57)	
Pf4 (1.56)	
Cycs (1.55)	
**Extracellular matrix and/or genes associated with fibrosis**	
Postn (3.17)	Cyr61 (3.57)
Col3a1 (2.76)	Ctgf (1.96)
Olfml2b (2.62)	Spock2 (1.52)
Lgals3 (2.55)	
Sulf2 (1.79)	
Col1a2 (1.73)	
Smoc2 (1.69)	
Col5a2 (1.67)	
Egflam (1.64)	
Col1a2 (1.64)	
Col1a1 (1.53)	
Crispld2 (1.50)	
Col5a1 (1.50)	
Ndufs4 (1.50)	
**Cell growth/proliferation**	
Cxcl12 (2.05)	Tacc2 (1.72)
Nppa (1.87)	Bcl2l1 (1.54)
Slit2 (1.78)	Xrcc5 (1.52)
Pla2g4a (1.73)	
Myocd (1.63)	
Sfrp1 (1.62)	
Bcl2 (1.62)	
Fanca (1.62)	
Atf5 (1.57)	
Emp1 (1.53)	
Serpine2 (1.52)	
Nf2 (1.52)	
Cgrrf1 (1.50)	
**Heart development/embryonic skeletal system development/actin cytoskeleton organization/multicellular organismal development**	
Wif1 (2.47)	Sorbs1 (1.82)
Dnase2a (2.11)	Enah (1.67)
Eda2r (2.04)	Dmd (1.61)
Pdlim7 (1.89)	Vamp5 (1.59)
Sulf2 (1.79)	Flnb (1.56)
Slit2 (1.78)	
Sema3c (1.74)	
Myocd (1.63)	
Sfrp1 (1.62)	
Bves (1.61)	
Ptpla (1.60)	
Cap1 (1.54)	
Sema3b (1.54)	
Col1a1 (1.53)	
Nf2 (1.52)	
Serpine2 (1.52)	
**Hypertrophy**	
Igf1 (1.85)	Lmcd1 (1.67)
**Immune/inflammatory response**	
Clec4n (2.14)	Plscr1 (1.89)
Cxcl12 (2.05)	Cblb (1.61)
Ccl8 (1.93)	
Slit2 (1.78)	
Ccl12 (1.71)	
Ly96 (1.67)	
C3ar1 (1.58)	
Ccl6 (1.57)	
Pf4 (1.56)	
Ccl9 (1.5)	
**Oxidation-reduction process**	
Cyp2b10 (4.00)	Cp (2.17)
Mthfd2 (3.13)	Fmo2 (1.92)
Pam (1.90)	Fdft1 (1.82)
Aldh1l2 (1.74)	Mical1 (1.72)
Asph (1.73)	D2hgdh (1.67)
Loxl2 (1.69)	Cyp2d22 (1.64)
Loxl1 (1.67)	P4htm (1.64)
Aldh18a1 (1.60)	Dmd (1.61)
Mecr (1.59)	Gpd1 (1.61)
Nqo2 (1.54)	Vamp5 (1.59)
Hsd17b7 (1.52)	Hsd17b10 (1.59)
Ndufs4 (1.50)	Bckdhb (1.56)
	Glrx3 (1.56)
	Cat (1.54)
	Fmo5 (1.52)
	Tet2 (1.52)

## References

[b1] Ferreira A. L., Matsubara L. S. & Matsubara B. B. Anthracycline-induced cardiotoxicity. Cardiovascular & hematological agents in medicinal chemistry 6, 278–281 (2008). 18855640 10.2174/187152508785909474

[b2] Lotrionte M. *et al.* Review and meta-analysis of incidence and clinical predictors of anthracycline cardiotoxicity. The American journal of cardiology 112, 1980–1984 (2013). 24075281 10.1016/j.amjcard.2013.08.026

[b3] Singal P. K., Li T., Kumar D., Danelisen I. & Iliskovic N. Adriamycin-induced heart failure: mechanism and modulation. Molecular and cellular biochemistry 207, 77–86 (2000). 10888230 10.1023/a:1007094214460

[b4] Kim Y. K. *et al.* Deletion of the inducible 70-kDa heat shock protein genes in mice impairs cardiac contractile function and calcium handling associated with hypertrophy. Circulation 113, 2589–2597 (2006). 16735677 10.1161/CIRCULATIONAHA.105.598409

[b5] Fan G. C. *et al.* Heat shock protein 20 interacting with phosphorylated Akt reduces doxorubicin-triggered oxidative stress and cardiotoxicity. Circulation research 103, 1270–1279 (2008). 18948619 10.1161/CIRCRESAHA.108.182832 PMC2763388

[b6] Wang X. *et al.* Overexpression of Hsp20 prevents endotoxin-induced myocardial dysfunction and apoptosis via inhibition of NF-kappaB activation. Journal of molecular and cellular cardiology 47, 382–390 (2009). 19501592 10.1016/j.yjmcc.2009.05.016 PMC2746739

[b7] Ballinger C. A. *et al.* Identification of CHIP, a novel tetratricopeptide repeat-containing protein that interacts with heat shock proteins and negatively regulates chaperone functions. Mol Cell Biol 19, 4535–4545 (1999). 10330192 10.1128/mcb.19.6.4535 PMC104411

[b8] Esser C., Scheffner M. & Hohfeld J. The chaperone-associated ubiquitin ligase CHIP is able to target p53 for proteasomal degradation. J Biol Chem 280, 27443–27448 (2005). 15911628 10.1074/jbc.M501574200

[b9] Meacham G. C., Patterson C., Zhang W., Younger J. M. & Cyr D. M. The Hsc70 co-chaperone CHIP targets immature CFTR for proteasomal degradation. Nat Cell Biol 3, 100–105 (2001). 11146634 10.1038/35050509

[b10] Hwang J. R., Zhang C. & Patterson C. C-terminus of heat shock protein 70-interacting protein facilitates degradation of apoptosis signal-regulating kinase 1 and inhibits apoptosis signal-regulating kinase 1-dependent apoptosis. Cell stress & chaperones 10, 147–156 (2005). 16038411 10.1379/CSC-90R.1 PMC1176473

[b11] Dickey C. A. *et al.* Akt and CHIP coregulate tau degradation through coordinated interactions. Proc Natl Acad Sci USA 105, 3622–3627 (2008). 18292230 10.1073/pnas.0709180105 PMC2265134

[b12] Zhou P. *et al.* ErbB2 degradation mediated by the co-chaperone protein CHIP. J Biol Chem 278, 13829–13837 (2003). 12574167 10.1074/jbc.M209640200

[b13] Xie P. *et al.* CHIP represses myocardin-induced smooth muscle cell differentiation via ubiquitin-mediated proteasomal degradation. Mol Cell Biol 29, 2398–2408 (2009). 19237536 10.1128/MCB.01737-08 PMC2668377

[b14] Dai Q. *et al.* CHIP activates HSF1 and confers protection against apoptosis and cellular stress. Embo J 22, 5446–5458 (2003). 14532117 10.1093/emboj/cdg529 PMC213783

[b15] Zhang C., Xu Z., He X. R., Michael L. H. & Patterson C. CHIP, a cochaperone/ubiquitin ligase that regulates protein quality control, is required for maximal cardioprotection after myocardial infarction in mice. Am J Physiol Heart Circ Physiol 288, H2836–2842 (2005). 15665051 10.1152/ajpheart.01122.2004

[b16] Naito A. T. *et al.* Promotion of CHIP-mediated p53 degradation protects the heart from ischemic injury. Circulation research 106, 1692–1702 (2010). 20413784 10.1161/CIRCRESAHA.109.214346

[b17] Xu C. W., Zhang T. P., Wang H. X., Yang H. & Li H. H. CHIP enhances angiogenesis and restores cardiac function after infarction in transgenic mice. Cellular physiology and biochemistry : international journal of experimental cellular physiology, biochemistry, and pharmacology 31, 199–208 (2013). 10.1159/000343361 23485987

[b18] Yang K. *et al.* Carboxyl terminus of heat shock protein 70-interacting protein inhibits angiotensin II-induced cardiac remodeling. American journal of hypertension 25, 994–1001 (2012). 22717542 10.1038/ajh.2012.74

[b19] Rose B. A., Force T. & Wang Y. Mitogen-activated protein kinase signaling in the heart: angels versus demons in a heart-breaking tale. Physiological reviews 90, 1507–1546. 20959622 10.1152/physrev.00054.2009 PMC3808831

[b20] Naito A. T. *et al.* Promotion of CHIP-mediated p53 degradation protects the heart from ischemic injury. Circulation research 106, 1692–1702 (2010). 20413784 10.1161/CIRCRESAHA.109.214346

[b21] Han Y. *et al.* Loss of SHP-1 enhances JAK3/STAT3 signaling and decreases proteosome degradation of JAK3 and NPM-ALK in ALK+ anaplastic large-cell lymphoma. Blood 108, 2796–2803 (2006). 16825495 10.1182/blood-2006-04-017434

[b22] Palen D. I., Belmadani S., Lucchesi P. A. & Matrougui K. Role of SHP-1, Kv.1.2, and cGMP in nitric oxide-induced ERK1/2 MAP kinase dephosphorylation in rat vascular smooth muscle cells. Cardiovasc Res 68, 268–277 (2005). 15967421 10.1016/j.cardiores.2005.05.031

[b23] Octavia Y. *et al.* Doxorubicin-induced cardiomyopathy: from molecular mechanisms to therapeutic strategies. Journal of molecular and cellular cardiology 52, 1213–1225 (2012). 22465037 10.1016/j.yjmcc.2012.03.006

[b24] Liu X. *et al.* Pifithrin-alpha protects against doxorubicin-induced apoptosis and acute cardiotoxicity in mice. Am J Physiol Heart Circ Physiol 286, H933–939 (2004). 14766674 10.1152/ajpheart.00759.2003

[b25] Toldo S. *et al.* Comparative cardiac toxicity of anthracyclines *in vitro* and *in vivo* in the mouse. PloS one 8, e58421 (2013). 23516478 10.1371/journal.pone.0058421 PMC3597611

[b26] Yen H. C., Oberley T. D., Vichitbandha S. & Ho Y. S. St Clair, D. K. The protective role of manganese superoxide dismutase against adriamycin-induced acute cardiac toxicity in transgenic mice. The Journal of clinical investigation 98, 1253–1260 (1996). 8787689 10.1172/JCI118909 PMC507548

[b27] Kang Y. J., Chen Y. & Epstein P. N. Suppression of doxorubicin cardiotoxicity by overexpression of catalase in the heart of transgenic mice. J Biol Chem 271, 12610–12616 (1996). 8647872 10.1074/jbc.271.21.12610

[b28] Greupink R. *et al.* The antiproliferative drug doxorubicin inhibits liver fibrosis in bile duct-ligated rats and can be selectively delivered to hepatic stellate cells *in vivo*. The Journal of pharmacology and experimental therapeutics 317, 514–521 (2006). 16439617 10.1124/jpet.105.099499

[b29] Zhu W., Shou W., Payne R. M., Caldwell R. & Field L. J. A mouse model for juvenile doxorubicin-induced cardiac dysfunction. Pediatric research 64, 488–494 (2008). 18614963 10.1203/PDR.0b013e318184d732 PMC2801890

[b30] Li L. *et al.* Preventive effect of erythropoietin on cardiac dysfunction in doxorubicin-induced cardiomyopathy. Circulation 113, 535–543 (2006). 16449733 10.1161/CIRCULATIONAHA.105.568402

[b31] Ito H. *et al.* Doxorubicin selectively inhibits muscle gene expression in cardiac muscle cells *in vivo* and *in vitro*. Proc Natl Acad Sci USA 87, 4275–4279 (1990). 2349236 10.1073/pnas.87.11.4275 PMC54091

[b32] Pashkow F. J. Oxidative Stress and Inflammation in Heart Disease: Do Antioxidants Have a Role in Treatment and/or Prevention? International journal of inflammation 31, 556–559 (2006). 10.4061/2011/514623 PMC3157078 21860805

[b33] Shizukuda Y., Matoba S., Mian O. Y., Nguyen T. & Hwang P. M. Targeted disruption of p53 attenuates doxorubicin-induced cardiac toxicity in mice. Molecular and cellular biochemistry 273, 25–32 (2005). 16013437 10.1007/s11010-005-5905-8

[b34] Li L. *et al.* Granulocyte colony-stimulating factor improves left ventricular function of doxorubicin-induced cardiomyopathy. Laboratory investigation; a journal of technical methods and pathology 87, 440–455 (2007). 10.1038/labinvest.3700530 17334414

[b35] Dickey C. A. *et al.* The high-affinity HSP90-CHIP complex recognizes and selectively degrades phosphorylated tau client proteins. The Journal of clinical investigation 117, 648–658 (2007). 17304350 10.1172/JCI29715 PMC1794119

[b36] Piatelli M. J., Doughty C. & Chiles T. C. Requirement for a hsp90 chaperone-dependent MEK1/2-ERK pathway for B cell antigen receptor-induced cyclin D2 expression in mature B lymphocytes. The Journal of biological chemistry 277, 12144–12150 (2002). 11823472 10.1074/jbc.M200102200

[b37] Maruyama T. *et al.* CHIP-dependent termination of MEKK2 regulates temporal ERK activation required for proper hyperosmotic response. The EMBO journal 29, 2501–2514 (2010). 20588253 10.1038/emboj.2010.141 PMC2928693

[b38] Calvisi D. F., Pascale R. M. & Feo F. Dissection of signal transduction pathways as a tool for the development of targeted therapies of hepatocellular carcinoma. Reviews on recent clinical trials 2, 217–236 (2007). 18474008 10.2174/157488707781662715

[b39] Hatakeyama S. Ubiquitin-mediated regulation of JAK-STAT signaling in embryonic stem cells. Jak-Stat 1, 168–175 (2012). 24058766 10.4161/jkst.21560 PMC3670240

[b40] Kolosenko I., Grander D. & Tamm K. P. IL-6 activated JAK/STAT3 pathway and sensitivity to Hsp90 inhibitors in multiple myeloma. Current medicinal chemistry 21, 3042–3047 (2014). 24735367 10.2174/0929867321666140414100831

[b41] LaFave L. M. & Levine R. L. JAK2 the future: therapeutic strategies for JAK-dependent malignancies. Trends in pharmacological sciences 33, 574–582 (2012). 22995223 10.1016/j.tips.2012.08.005

[b42] Kunisada K. *et al.* Signal transducer and activator of transcription 3 in the heart transduces not only a hypertrophic signal but a protective signal against doxorubicin-induced cardiomyopathy. Proc Natl Acad Sci USA 97, 315–319 (2000). 10618415 10.1073/pnas.97.1.315 PMC26660

[b43] Klingmuller U., Lorenz U., Cantley L. C., Neel B. G. & Lodish H. F. Specific recruitment of SH-PTP1 to the erythropoietin receptor causes inactivation of JAK2 and termination of proliferative signals. Cell 80, 729–738 (1995). 7889566 10.1016/0092-8674(95)90351-8

[b44] Zhang J., Somani A. K. & Siminovitch K. A. Roles of the SHP-1 tyrosine phosphatase in the negative regulation of cell signalling. Seminars in immunology 12, 361–378 (2000). 10995583 10.1006/smim.2000.0223

[b45] Forget G., Gregory D. J., Whitcombe L. A. & Olivier M. Role of host protein tyrosine phosphatase SHP-1 in Leishmania donovani-induced inhibition of nitric oxide production. Infect Immun 74, 6272–6279 (2006). 17057094 10.1128/IAI.00853-05 PMC1695482

[b46] An H. *et al.* Phosphatase SHP-1 promotes TLR- and RIG-I-activated production of type I interferon by inhibiting the kinase IRAK1. Nat Immunol 9, 542–550 (2008). 18391954 10.1038/ni.1604

[b47] Li H. H. *et al.* Atrogin-1/muscle atrophy F-box inhibits calcineurin-dependent cardiac hypertrophy by participating in an SCF ubiquitin ligase complex. The Journal of clinical investigation 114, 1058–1071 (2004). 15489953 10.1172/JCI22220 PMC522252

[b48] Wang L. *et al.* Inhibition of Toll-like receptor 2 reduces cardiac fibrosis by attenuating macrophage-mediated inflammation. Cardiovasc Res 101, 383–392 (2014). 24259498 10.1093/cvr/cvt258

[b49] Yang D., Guo S., Zhang T. & Li H. Hypothermia attenuates ischemia/reperfusion-induced endothelial cell apoptosis via alterations in apoptotic pathways and JNK signaling. FEBS letters 583, 2500–2506 (2009). 19596001 10.1016/j.febslet.2009.07.006

[b50] Yang D., Xie P., Guo S. & Li H. Induction of MAPK phosphatase-1 by hypothermia inhibits TNF-alpha-induced endothelial barrier dysfunction and apoptosis. Cardiovasc Res 85, 520–529 (2010). 19793766 10.1093/cvr/cvp323

[b51] Wei S. N. *et al.* Microarray and Co-expression Network Analysis of Genes Associated with Acute Doxorubicin Cardiomyopathy in Mice. Cardiovascular toxicology 15, 377–393 (2015). 25575753 10.1007/s12012-014-9306-7

[b52] Bonnes-Taourel D., Guerin M. C. & Torreilles J. Is malonaldehyde a valuable indicator of lipid peroxidation? Biochemical pharmacology 44, 985–988 (1992). 1530665 10.1016/0006-2952(92)90132-3

[b53] Brazma A. *et al.* Minimum information about a microarray experiment (MIAME)-toward standards for microarray data. Nature genetics 29, 365–371 (2001). 11726920 10.1038/ng1201-365

[b54] Zhao W. J. *et al.* Gene expression profiling identifies the novel role of immunoproteasome in doxorubicin-induced cardiotoxicity. Toxicology 333, 76–88 (2015). 25896364 10.1016/j.tox.2015.04.009

[b55] Wajima Z., Shiga T., Imanaga K. & Inoue T. Vigilance of hemodynamic changes immediately after transferring patients is crucial. Journal of anesthesia 27, 521–527 (2013). 23455699 10.1007/s00540-013-1568-x

