# Supplementary material for: Protection against doxorubicin-induced myocardial dysfunction in mice by cardiac-specific expression of carboxyl terminus of hsp70-interacting protein
Source: Sci Rep. 2016 Jun 21;6:28399. doi: 10.1038/srep28399 (PMC4914971; doi:10.1038/srep28399)
Supplement: Supplementary Information [file srep28399-s1.pdf]

Protection against doxorubicin-induced myocardial dysfunction in mice by cardiac-specific expression of carboxyl terminus of hsp70-interacting protein

Lei Wang[1], Tian-Peng Zhang[1], Yuan Zhang[2], Hai-Lian Bi[1], Xu-Min Guan[1], Hong-Xia Wang[3], Xia Wang[3], Jie Du[4], Yun-Long Xia[1], Hui-Hua Li[1,5*]

Supplementary Materials

Table S1. Primers used for Quantitative real-time PCR

Gene	Forward Primer	Reverse Primer
IL-1β	5’-CTTCCCCAGGGCATGTTAAG-3’	5’-ACCCTGAGCGACCTGTCTTG-3’
IL-6	5’-TTCCATCCAGTTGCCTTCTTG-3’	5’-TTGGGAGTGGTATCCTCTGTGA-3’
Bax	5’-AAACTGGTGCTCAAGGCCC-3’	5’- CTTGGATCCAGACAAGCAGC-3’
Bcl-2	5’-GGAGGCTGGGATGCCTTTGT-3’	5’-TGCACCCAGAGTGATGCAG-3’
TNF-α	5’-ATGGCCTCCCTCTCATCAGT-3’	5’-CTTGGTGGTTTGCTACGACG-3’
Cyp2b10	5’-TCCTCAAGTCTTTTATTCAGCTTCG-3’	5’-TGAAGGTTGGCTCAACGACA-3’
Mthfd2	5’-CCAGCGCTACTTGGTGACTG-3’	5’-ACAACGGCTTCATTTCTGGTG-3’
Postn	5’-GAAGTGATCCACGGAGAGCC-3’	5’-TGTTTCTCCACCTCCTGTGG-3’
Col3a1	5’-TGACTGTCCCACGTAAGCAC-3’	5’-GAGGGCCATAGCTGAACTGA-3’
Cyr61	5’-AGAGGCTTCCTGTCTTTGGC-3’	5’-CTCGTGTGGAGATGCCAGTT-3’
Btg2	5’-CGCACTGACCGATCATTACAA-3’	5’-GGATCAACCCACAGGGTCAG-3’
Lcn2	5’-CCCTGTATGGAAGAACCAAGGA-3’	5’-CCACACTCACCACCCATTCA-3’
Igf1	5’-TCTGCCTCTGTGACTTCTTGA-3’	5’-TAGCCTGTGGGCTTGTTGAA-3’
GAPDH	5’-GGTTGTCTCCTGCGACTTCA-3’	5’-GGTGGTCCAGGGTTTCTTACTC-3’

IL-1β, Interleukin-1β; IL-6, Interleukin-6; TNF-α, Tumor necrosis factor ; Cyp2b10, cytochrome P450 family 2 subfamily b polypeptide 10; Mthfd2, methylenetetrahydrofolate dehydrogenase (NAD+ dependent) methenyltetrahydrofolate cyclohydrolase; Postn, periostin osteoblast specific factor; Col3a1, collagen type III alpha 1; Btg2, B-cell translocation gene 2 anti-proliferative; Igf1, insulin-like growth factor 1; Cyr61, cysteine rich protein 61; Lcn2, lipocalin 2; GAPDH, Glyceraldehyde 3-phosphate dehydrogenase.

Materials and methods

Antibodies and reagents. Antibodies including anti-CHIP and anti-p53 (Santa Cruz), anti-AKT, anti–phospho-AKT (Ser473), anti-Jak, anti-phospho-Jak, anti-JNK1/2, anti-phospho-JNK1/2, anti-ERK1/2, anti-phospho-ERK1/2 (Thr202/Tyr204), anti-signal transducer and activator of transcription 3 (STAT3, Tyr705), anti-phospho-STAT3, anti-β-actin, SHP1, MKP1, GAPDH and anti-rabbit or anti-mouse antibody horseradish peroxidase-linked IgG were purchased from Cell Signaling Technology (Beverly, MA). Anti-Mac-2 (CD11b) antibody was from Abcam, Inc. (Cambridge, UK).

Echocardiography. Mice were anesthetized with 1.5% isoflurane by inhalation. Left ventricular internal dimensions at end-diastole (LVEDD), left ventricular posterior wall thickness at diastole (LVPWD), left ventricular anterior wall thickness at diastole (LVAWD), fractional shortening (FS) and ejection fraction (EF) were measured digitally on the M-mode tracings and averaged from at least 3 separate cardiac cycles.

RNA analysis. Hearts from WT and CHIP-TG mice were excised, rinsed in PBS, frozen in liquid nitrogen. Total RNA was extracted by the Trizol reagent method (Invitrogen). The RNA purity was detected by the absorbance value of 260/280 (1.8-2.0). The first strand cDNA was synthesized with Moloney murine leukaemia virus reverse transcriptase (Promega, Southampton, UK). The levels of cytochrome P450 family 2 subfamily b polypeptide 10 (Cyp2b10), methylenetetrahydrofolate dehydrogenase (NAD+ dependent) methenyltetrahydrofolate cyclohydrolase (Mthfd2), periostin osteoblast specific factor (Postn), collagen type III alpha 1 (Col3a1), B-cell translocation gene 2 anti-proliferative (Btg2), insulin-like growth factor 1 (Igf1), cysteine rich protein 61 (Cyr61) and lipocalin 2 (Lcn2) mRNA expression were measured by quantitative real-time PCR (qPCR) with an iCyclerIQ system (Bio-Rad, USA).[20]

Western blot analysis. The blots were developed by use of a chemiluminescent system, and densitometry analysis involved a Gel-pro 4.5 Analyzer (Media Cybernetics, USA). Relative protein levels were normalized to that of β-actin.

Microarray Gene Expression Analysis. Total RNA was extracted with Trizol reagent (Invitrogen) from the hearts of saline/DOX-treated WT and CHIP TG mice (n = 3 per group) at day 5 after injection. The mRNA samples were prepared for hybridizing to Affymetrix Gene Chip mouse Genome 430 2.0 array and scanned in Affymetrix Gene Chip Confocal Scanner 3000 according to the protocols as described previously.[24] Both experimental design and comprehensive bioinformatics analysis were done in compliance with the Minimum Information About a Microarray Experiment guidelines.[25] The random-variance model (RVM) F-test was applied to filter the differentially expressed genes for the each group. We selected the differentially expressed genes according to the P-value threshold.[21]

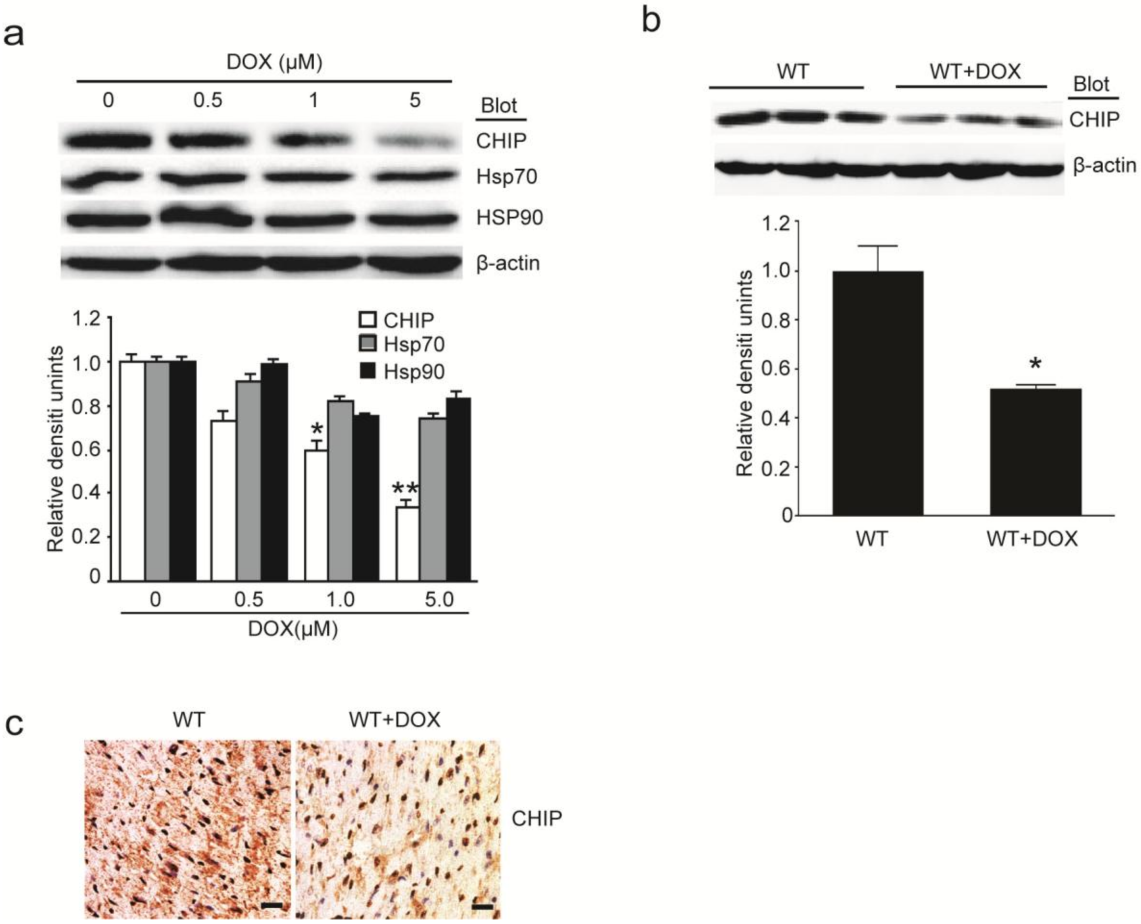

Supplemental Figure 1. Doxorubincin treatment down-regulates CHIP expression in neonatal rat cardiomyocytes and the mouse hearts. (a) Cardiomyocytes were treated with different doses of DOX at 0.5，1, or 5 μM for 24 hours. The levels of CHIP, HSP70, HSP90 and β-actin protein were detected by Western blot analysis (left). Quantitative analysis of protein bands (right). *P < 0.05, ** P < 0.01 versus 0 hour. **(b)** WT mice were injected with a single dose of 20 mg/kg DOX or saline for 5 days. The levels of CHIP and β-actin protein in mouse hearts were determined (left). Quantitative analysis of protein bands (right). *P < 0.05 versus WT+saline. **(c)** Heart sections from saline-treated and DOX-treated mice were immunostained with anti-CHIP antibody (scale bar = 50μm).

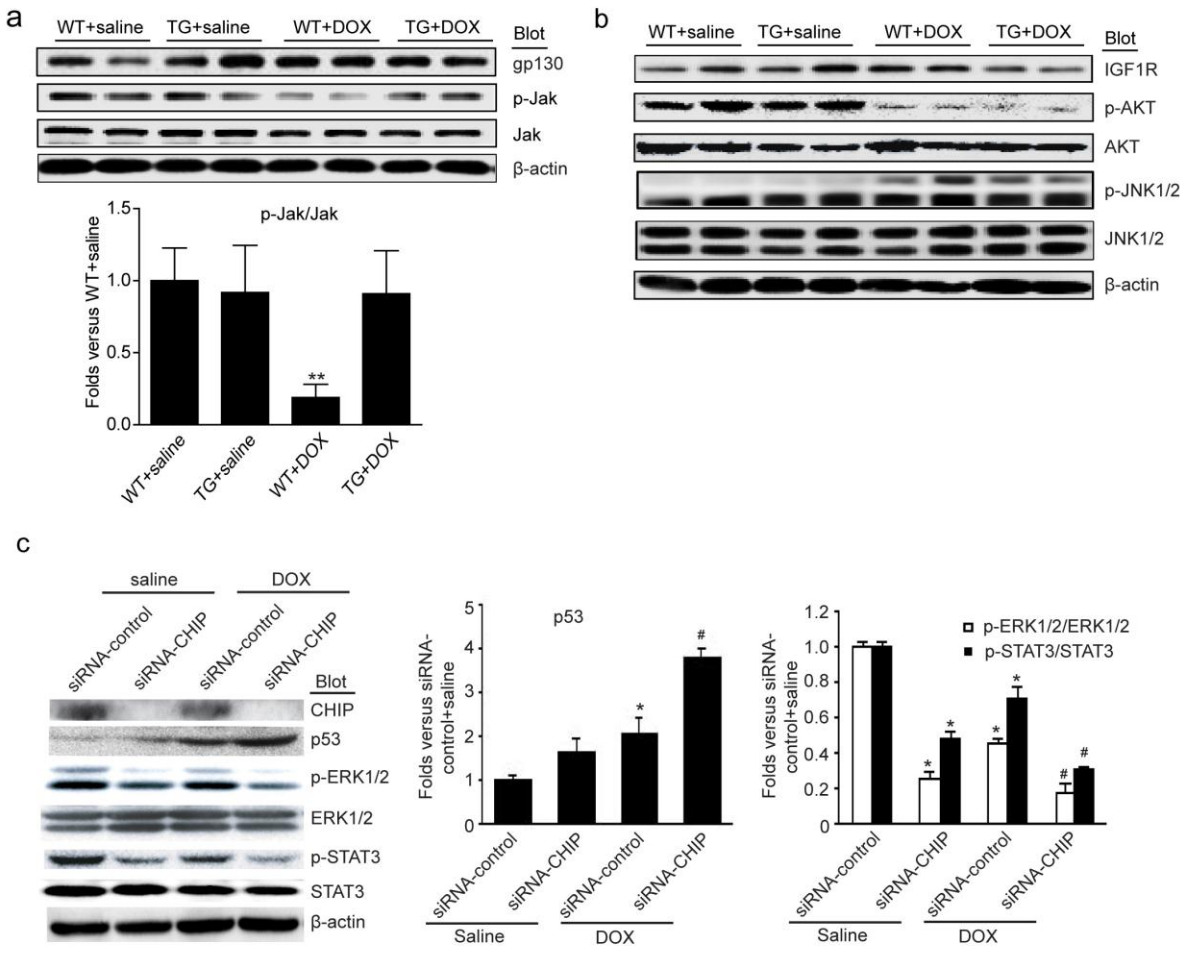

Supplemental Figure 2. Western blot analysis protein levels of gp130/Jak, IGF1R/AKT, JNK1/2 in the WT or CHIP-TG heart and p53, p-ERK1/2/ERK1/2 and p-STAT3/STAT3 protein levels in cardiomyocytes infected with Ad-siRNA-control or Ad-siRNA-CHIP. (a) Western blot analysis of protein levels of gp130 and p-Jak/ Jak from WT and CHIP-TG hearts. **(b)** Western blot analysis of protein levels of p-AKT/AKT and p-JNK1/2/JNK1/2 in the WT and CHIP-TG hearts. **(c)** Cardiomyocytes were infected with Ad-siRNA-control or Ad-siRNA-CHIP and treated with saline or DOX. Western blot analysis of p53, p-ERK1/2/ERK1/2 and p-STAT3 protein levels (left). Quantitative analysis of relative protein levels (right). *$P < 0.05$ versus siRNA-control+saline; $^{\#}P < 0.05$ versus siRNA-control+DOX.

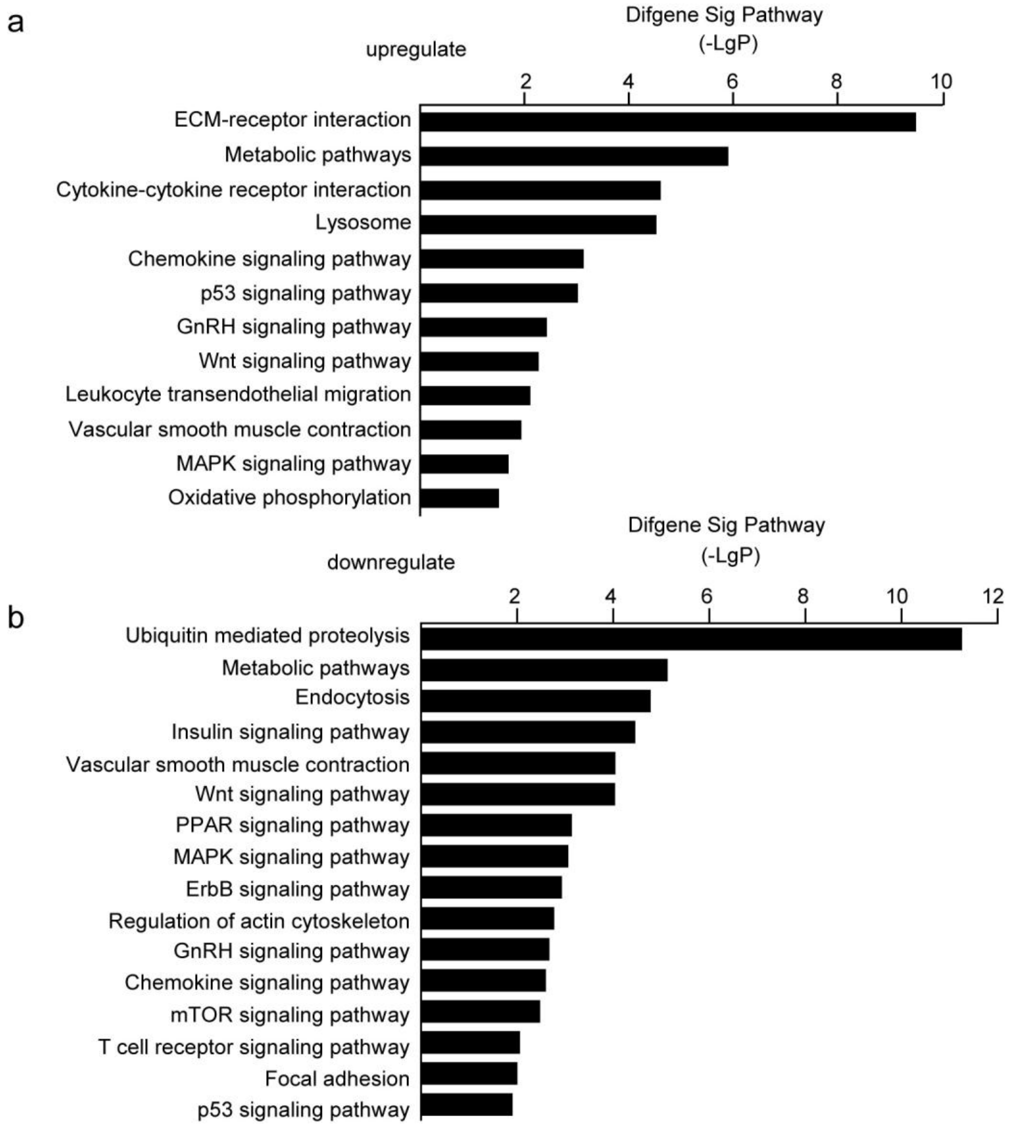

Supplemental Figure 3. Pathway analysis of genes in the heart of WT and CHIP-TG mice after DOX injection. Pathways of significant up-regulated **(a)** and down-regulated **(b)** genes were shown.
